# *Wnt5a* Regulates Ventral Midbrain Morphogenesis and the Development of A9–A10 Dopaminergic Cells *In Vivo*

**DOI:** 10.1371/journal.pone.0003517

**Published:** 2008-10-27

**Authors:** Emma R. Andersson, Nilima Prakash, Lukas Cajanek, Eleonora Minina, Vitezslav Bryja, Lenka Bryjova, Terry P. Yamaguchi, Anita C. Hall, Wolfgang Wurst, Ernest Arenas

**Affiliations:** 1 Laboratory of Molecular Neurobiology, Department of Medical Biochemistry & Biophysics, Karolinska Institutet, Stockholm, Sweden; 2 Helmholtz Centre Munich, German Research Centre for Environmental Health, and Technical University Munich, Institute of Developmental Genetics, Munich/Neuherberg, Germany; 3 Department of Cytokinetics, Institute of Biophysics, Academy of Sciences of the Czech Republic, Brno, Czech Republic; 4 Institute of Experimental Biology, Faculty of Science, Masaryk University, Brno, Czech Republic; 5 Cancer and Developmental Biology Laboratory, National Cancer Institute-Frederick, Frederick, Maryland, United States of America; 6 Division of Cell and Molecular Biology, Imperial College London, London, United Kingdom; 7 Max-Planck-Institute of Psychiatry, Munich, Germany; Katholieke Universiteit Leuven, Belgium

## Abstract

Wnt5a is a morphogen that activates the Wnt/planar cell polarity (PCP) pathway and serves multiple functions during development. PCP signaling controls the orientation of cells within an epithelial plane as well as convergent extension (CE) movements. *Wnt5a* was previously reported to promote differentiation of A9–10 dopaminergic (DA) precursors *in vitro*. However, the signaling mechanism in DA cells and the function of *Wnt5a* during midbrain development *in vivo* remains unclear. We hereby report that Wnt5a activated the GTPase Rac1 in DA cells and that Rac1 inhibitors blocked the Wnt5a-induced DA neuron differentiation of ventral midbrain (VM) precursor cultures, linking Wnt5a-induced differentiation with a known effector of Wnt/PCP signaling. *In vivo*, *Wnt5a* was expressed throughout the VM at embryonic day (E)9.5, and was restricted to the VM floor and basal plate by E11.5–E13.5. Analysis of *Wnt5a−/−* mice revealed a transient increase in progenitor proliferation at E11.5, and a precociously induced NR4A2+ (Nurr1) precursor pool at E12.5. The excess NR4A2+ precursors remained undifferentiated until E14.5, when a transient 25% increase in DA neurons was detected. *Wnt5a−/−* mice also displayed a defect in (mid)brain morphogenesis, including an impairment in midbrain elongation and a rounded ventricular cavity. Interestingly, these alterations affected mostly cells in the DA lineage. The ventral Sonic hedgehog-expressing domain was broadened and flattened, a typical CE phenotype, and the domains occupied by Ngn2+ DA progenitors, NR4A2+ DA precursors and TH+ DA neurons were rostrocaudally reduced and laterally expanded. In summary, we hereby describe a *Wnt5a* regulation of Wnt/PCP signaling in the DA lineage and provide evidence for multiple functions of Wnt5a in the VM *in vivo*, including the regulation of VM morphogenesis, DA progenitor cell division, and differentiation of NR4A2+ DA precursors.

## Introduction

Wnts comprise a family of 19 lipid-modified secreted glycoproteins that signal via different pathways and regulate multiple aspects of development [1], [2]. These pathways include the canonical Wnt/β-catenin, noncanonical Wnt/Ca^2+^ and noncanonical Wnt/planar cell polarity (PCP) pathways.

Wnt5a has been reported to activate both canonical and noncanonical signaling depending on receptor, cellular and tissue context [3]–[5]. However, Wnt5a is generally considered a noncanonical Wnt that activates PCP or Ca^2+^ signaling [6]. Most PCP genes were initially identified in *Drosophila* or *Xenopus* and their homologues were subsequently found in mammals. These include genes for transmembrane proteins, such as *frizzled* (*fz/Fz*) [7]–[9], *Van Gogh/Strabismus* (*Vang/Vangl/stbm*) [10], [11], and *starry night/flamingo/Celsr* (*stan/fmi/Celsr*) [12], [13]. Some cytoplasmic components of this pathway are shared with the Wnt/β-catenin pathway, such as *Dishevelled* (*dsh/Dvl*) [14], [15] and *Casein kinase 1* (*Ck1*) [16]–[18]. Specific Wnt/PCP cytoplasmic components include *Daam*, small GTPases of the Rho family: *Cdc42*, *Rac1*, *RhoA*, the *Rho kinase* and *JNK* [19]–[22].

Mutations in PCP genes produce specific and distinctive phenotypes. These include the general convergent extension (CE) defects seen in the overall shortened and broadened morphology of mutants and in Keller explants of the *Xenopus* dorsal marginal zone [23], [24], the *Drosophila* wing with misdirected bristles and disorganization of the compound eye [2], [25], the murine cochlea with misdirected hair cells [4], [26], and the murine neural tube with a broadened Shh-expressing floor plate (FP) and neural tube closure defects [9], [27], [28]. PCP is defined as the organization of cells within a single layered sheet of cells. CE however, is a morphological process regulated by PCP signaling and involving the coordinated movement of cells within a 3-dimensional structure, leading to an overall elongation and narrowing of the structure [24]. Moreover, a role for PCP genes in the development of region-specific neuronal cell types outside of an epithelial plane is becoming increasingly apparent. For instance, PCP genes *Celsr3* and *Frizzled3* are involved in axon growth and guidance, while *Celsr1* and *Dvl1* also regulate dendritic arborization (for review see [29]).

In zebrafish, the *Wnt5* and *Wnt11* mutants, *pipetail* and *silberblick* respectively, display CE defects but their roles have been suggested to be more permissive than instructive [30], [31]. However, *Wnt5a* has been found to be required for stereocilia orientation in the cochlea, indicating that Wnt5a can play an instructive role in PCP during development [4]. We hypothesized that Wnt5a may be able to activate PCP signaling and contribute to patterning and subsequent neural differentiation in specific brain regions.

We have previously reported that Wnt5a promotes DA differentiation of NR4A2 precursors in primary mesencephalic cultures [32]. However, the function of *Wnt5a* in the developing VM *in vivo* is unknown. A9–A10 dopaminergic neurons of the substantia nigra (SN) and ventral tegmental area (VTA) respectively, are born between E10.5 and E13.5. We therefore set out to investigate whether deletion of *Wnt5a* results in a Wnt/PCP phenotype and/or deficits in NR4A2 precursor differentiation *in vivo* at these and later stages up to E18.5. We provide evidence that *Wnt5a* is required for adequate morphogenesis of the midbrain by controlling the proper polarity and proliferation of VM progenitors, and for the differentiation of postmitotic NR4A2+ precursors into DA neurons.

## Results

### Wnt5a is expressed in a restricted temporal and spatial pattern during midbrain development

**Figure 1:**
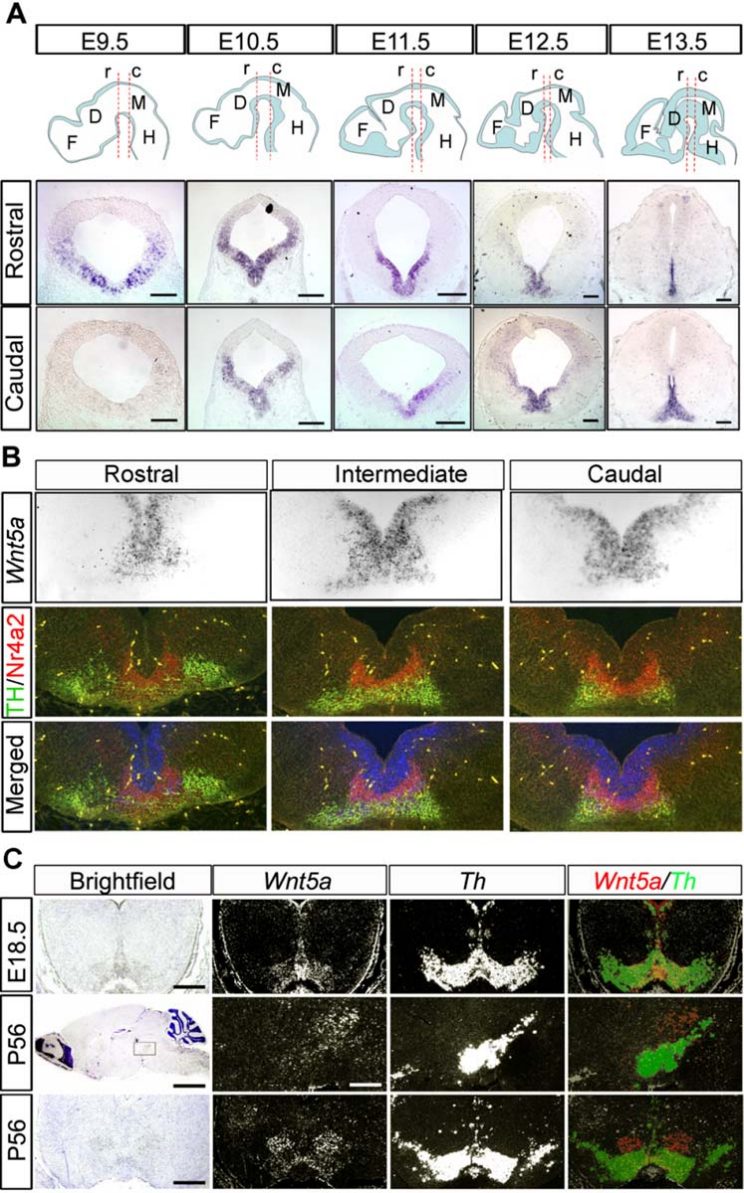
Temporal and spatial expression of *Wnt5a* in the mouse. (A) In situ hybridization for *Wnt5a* on coronal midbrain sections of E9.5–E13.5 CD1 mice shows a dynamic regulation of *Wnt5a* expression domains throughout development in both rostrocaudal distribution and developmental stages. Schemes with sagittal sections of the brain and dashed lines show the levels at which rostral (r) or caudal (c) expression analysis was performed. The expression of *Wnt5a* occupies the entire neuroepithelium of the ventral midbrain at E9.5 and 10.5, and becomes progressively restricted to the floor plate and basal plate ventricular zone from E11.5–E13.5, extending to the marginal zone at caudal levels (Scale bars at E9.5 = 100 µm, at E10.5 and E11.5 = 200 µm, at E12.5 and E13.5 = 175 µm). (B) At E12.5, the expression of *Wnt5a* comprises the FP extending from the ventricular zone through the intermediate zone and into the marginal zone, overlapping with NR4A2+ and TH+ cells. This extension into the marginal zone is most pronounced in the caudal midbrain. (C) The expression of *Wnt5a* is down-regulated by E18.5, where it still overlaps with TH+ cells, but 8 weeks after birth, at P56, this overlap is lost as seen in sagittal and coronal sections. (Scale bars in brightfield E18.5, coronal P56 and darkfield sagital p56 = 500 µm, scale bar in brightfield sagital P56 = 2.5 mm). Abbreviations: r = rostral, c = caudal, F = forebrain, D = diencephalon, M = midbrain, H = hindbrain.

We have previously reported that *Wnt5a* is highly expressed in the VM at the time of birth of DA neurons [32]. To examine the spatial and temporal expression of *Wnt5a* more closely we performed in situ hybridization on E9.5–E13.5, E18.5 and P56 CD1 mice. Interestingly, in situ hybridization showed a gradient of *Wnt5a* expression, with higher expression of *Wnt5a* at E9.5 and 10.5 in rostral levels of VM as compared to caudal levels (Figure 1A). From E11.5 to E13.5, the expression of *Wnt5a* became progressively restricted to the FP and basal plate (BP). Until E11.5 *Wnt5a* was only expressed in the ventricular zone (VZ)/subventricular zone (SVZ), but from E12.5, and mainly in caudal levels, *Wnt5a* was found in the intermediate and marginal zones, extending laterally (Figure 1A). We next examined the expression of *Wnt5a* in relation to *tyrosine hydroxylase* (*TH* - the rate-limiting enzyme in the synthesis of dopamine and a marker of DA neurons) expression. Double immunohistochemistry for TH and NR4A2 (a marker of DA neurons and precursors) on sections probed for *Wnt5a* revealed extensive overlap of expression, particularly in the caudal midbrain at E12.5 (Figure 1B and data not shown for other stages). From E13.5 on, the expression of *Wnt5a* in the ventricular zone (VZ) was reduced, and at E18.5, it became mostly restricted to DA neurons of the ventral tegmental area (VTA) (Figure 1C). In the postnatal VM, expression of *Wnt5a* was not detected in substantia nigra compacta (SNc) neurons, was faintly detectable in VTA neurons, and was mainly confined to the red nucleus (Figure 1C).

### Wnt5a activates Rac1, and inhibition of Rac1 blocks Wnt5a-induced DA neurogenesis

**Figure 2:**
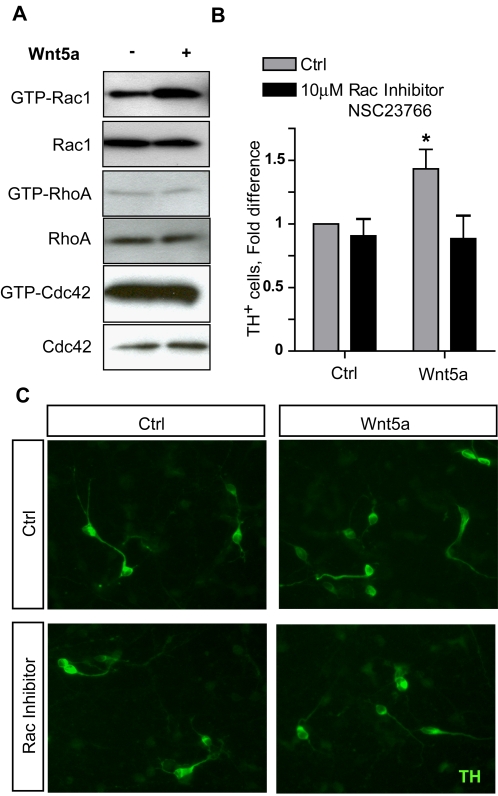
Rac1 is activated by Wnt5a and is required for Wnt5a-induced dopaminergic neuron differentiation. (A) Stimulation of SN4741 cells, a midbrain dopaminergic neuron cell line, with 200 ng/ml Wnt5a for 2 hrs activates Rac1, but not RhoA or Cdc42. (B) As previously reported, Wnt5a upregulates the number of TH+ dopaminergic neurons in E11.5 primary ventral midbrain cultures grown for three days in vitro. This differentiation is completely inhibited in the presence of 10 µM NSC23766, a Rac1 inhibitor (ANOVA for Wnt5a vs Wnt5a&NSC23766, P<0.05, N = 3 experiments, each normalized to the control conditions) (C) TH+ cells in all conditions have normal morphology, with increased numbers in the Wnt5a-treated, but not Rac-inhibited, wells.

We have previously shown that Wnt5a promotes the differentiation of dopaminergic (DA) neurons in primary midbrain cultures [32] and that Wnt5a signals via Dishevelled and Casein Kinase 1 in a dopaminergic cell line [17], [33]. Wnt5a is known to activate the PCP pathway and to signal via small GTPases in different systems [34], but it is unknown which of the small GTPases transduces the Wnt5a signal in cells of the DA lineage. We therefore first investigated whether Wnt5a could activate Rac1, RhoA or Cdc42 in a DA cell line, SN4741, a validated model for studying Wnt signaling [33], [35]–[37]. We found that treatment with recombinant mouse Wnt5a induced the activation of Rac1 (Figure 2A), while RhoA and cdc42 activity were unchanged. In order to verify whether Rac1 mediates the pro-differentiation effects of Wnt5a, we used an *in vitro* assay in which Wnt5a induces the differentiation of primary DA precursors into DA neurons [32]. A dose-response curve for NSC 23766, a Rac inhibitor, showed that 10 µM had no effect on TH+ cell number. At higher doses, from 50 µM and up, cell death was seen (data not shown). Interestingly, treatment of these cultures with 10 µM of the Rac1 inhibitor NSC 23766 blocked the increase in the number of TH+ neurons otherwise induced by Wnt5a after 3 days *in vitro* (Figure 2 B, C), suggesting that the pro-differentiation effects of Wnt5a are mediated by Rac1. Note that the morphology of the TH+ cells is unchanged, and that the cells appear healthy in all conditions (Figure 2C).

In the next part of our study we analyzed the VM phenotype of *Wnt5a−/−* mice generated previously [38]. We have previously reported that Wnt5a does not activate canonical signaling in a dopaminergic neuron cell line [33]. Data in the literature suggests that Wnt/β-catenin signaling could be decreased in *Wnt5a−/−* mice, since Wnt5a has been reported as capable of activating Wnt/β-catenin signaling [3], or increased since non-canonical signaling has also been reported to inhibit Wnt/β-catenin signaling [39]. To address possible regulation of canonical signaling in the *Wnt5a−/−* VM, we first examined the expression of Wnt1, a Wnt expressed in the VM that activates the Wnt/β-catenin pathway and is required for VM DA neuron development [40]. In situ hybridization did not show any increase in signal, but rather a wider spacing of the two ventral stripes of Wnt1 expression in the FP (Figure S1A). This was confirmed by QPCR analysis of E12.5 WT and *Wnt5a−/−* VMs tissue (Figure S1B), a finding that argues against a significant imbalance between Wnt1 and Wnt5a. To examine canonical signaling at the effector level, we investigated the levels of active β-catenin by Western blot in the VM of E10.5 and E12.5 *Wnt5a−/−* mice, where no difference was detected (Figure S1C and data not shown). In sum, Wnt5a activated the small GTPase Rac1 and promoted DA differentiation via Rac1, but loss of *Wnt5a* had no effect on Wnt1 expression or canonical Wnt-signaling via β-catenin, suggesting that Wnt5a may regulate Wnt/PCP signaling in the ventral midbrain *in vivo*.

### Differentiation of dopaminergic neurons is altered in *Wnt5a−/−* mice

**Figure 3:**
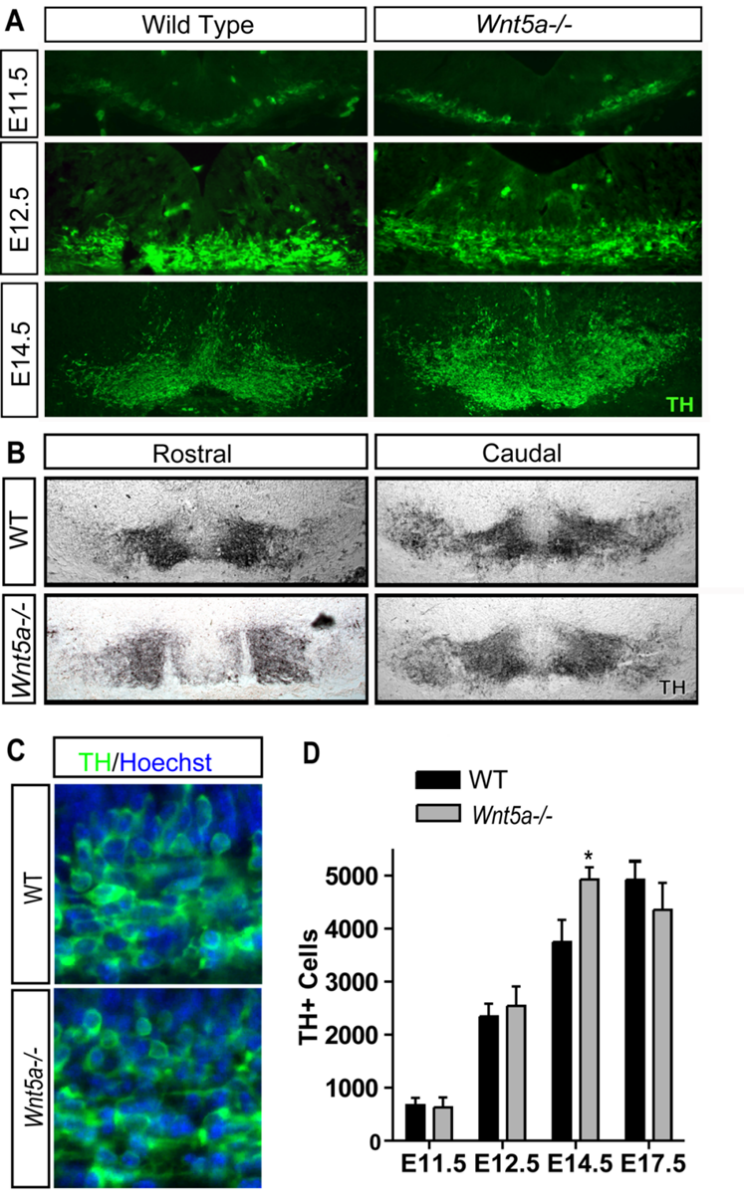
The number of dopaminergic neurons is normal at most stages but transiently increases at E14.5 in *Wnt5a*−/− mice. (A) At E11.5 and E12.5 no differences in the number of TH+ cells could be detected. At E12.5 and E14.5 the region occupied by TH+ cells in the *Wnt5a−/−*midbrain appears larger, extending both laterally and dorsally. (B) At E17.5 the distribution of cells is broader dorsoventrally in rostral and caudal sections in the *Wnt5a−/−* VM. Importantly, at rostral levels the ventral tegmental area was more lateral (leaving a TH-poor midline domain) and the substantia nigra more medial, making it difficult to differentiate between them. (C) Enlarged image of TH+ cells at E12.5 shows normal DA neuron morphology in the *Wnt5a−/−* mice. (D) Quantification of the number of TH+ cells in E11.5, E12.5, E14.5, and E17.5 mice shows a transient 25% increase at E14.5 in *Wnt5a−/−* embryos, which was no longer seen at E17.5. (At E14.5, unpaired t-test, p = 0.0492, N = 4).

**Figure 4:**
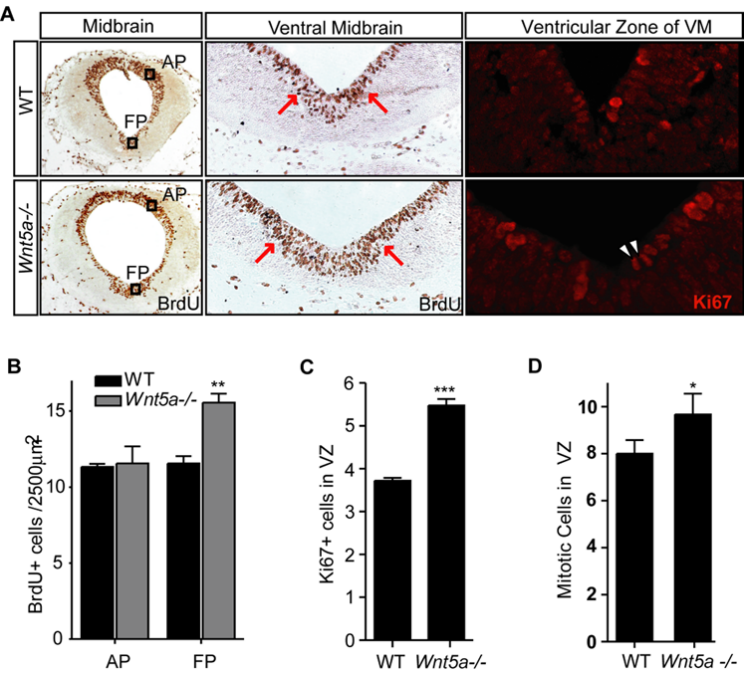
Increased proliferation and accumulation of progenitor cells in *Wnt5a−/−* mice. (A). Representative coronal sections at the level of the midbrain of 2 hr BrdU-pulsed WT and *Wnt5a−/−* embryos at E11.5 immunostained for BrdU or Ki67, markers of cells in mitosis. The black squares in (A) depict the area within the alar plate (AP) and floor plate (FP) used for the quantification of BrdU+ cells in (B) (Squares in (A) are not drawn to scale). Note the accumulation of BrdU+ cells in the *Wnt5a*−/− VM in the adjacent magnified box. Increased Ki67 staining and mitotic figures (arrowheads) in the ventricular zone. (B) The density of BrdU+ proliferating cells was significantly increased within the FP, but no significant change was found within the AP of *Wnt5a−/−* embryos compared to their *wt* littermates (C) The number of Ki67+ cells was significantly increased at E12.5 (paired t-test, p = 0.003, N = 3). (D) Mitotic nuclei (arrowheads in (A)) were counted in the midline domain to asses the number of cells in M-phase, and this was also found to be significantly increased in the *Wnt5a−/−* mutant (paired t-test, p = 0.037, N = 3).

To assess the role of Wnt5a *in vivo*, we examined the A9–A10 DA neuron populations in *Wnt5a* knockout mice at E11.5, E12.5, E14.5, E17.5 and E18.5. Surprisingly, at E11.5 and E12.5, the number of TH+ DA neurons in the *Wnt5a−/−* mice was not statistically different from wild-type (WT) littermate controls (Figure 3A, D). This was confirmed by quantitative PCR (QPCR) for TH and for Pitx3 (Figure S 2A,B), a homeobox transcription factor required for DA neuron survival [41]–[43]. A transient 25% increase in the number of Th+ DA neurons was detected at E14.5 (Figure 3A, D) but not at earlier or later stages (Figure 3B, D and data not shown). TH+ cells displayed normal morphology and relatively normal density at E12.5 (Figure 3C). Although the number of DA neurons normal at all ages except E14.5, the morphology of the midbrain and distribution of the DA neurons was altered in *Wnt5a−/−* mice from E10.5, note the broader lateral and dorsoventral distribution of TH+ cells at E12.5, E14.5 and E17.5 (Figure 4A, B). We also examined the number of cleaved caspase 3+ cells (a marker of cells in apoptosis), but found no difference at E14.5 or E17.5 (data not shown).

### Increased proliferation and DA precursor pool in *Wnt5a−/−* mice

*In vitro*, Wnt5a promotes DA precursor differentiation, reducing the number of proliferating (in S-phase) bromodeoxyuridine positive (BrdU+) cells [32]. To assess the role of Wnt5a in proliferation *in vivo* and the reason for the transient 25% increase in Th+ cells in the *Wnt5a−/−* embryos, the density of BrdU+ cells/area in the FP and the alar plate (AP) of E11.5 *Wnt5a−/−* and WT embryos were compared (black squares in Figure 4A). While the density of BrdU+ cells in the AP of WT and *Wnt5a−/−* embryos was not different (after a 2 hour pulse), a 35% increase in BrdU+ cells/area was detected in the FP of *Wnt5a−/−* mice (Figure 4A, B). Furthermore, BrdU+ cells accumulated in the ventral midline of the *Wnt5a−/−* midbrain at E11.5 (Figure 4A). The increase of proliferating cells within the FP was first evident at E11.5. This was confirmed by a 45% increase in Ki67+ cells (a marker of cells in the cell cycle) detected in *Wnt5a−/−* mice (Figure 4A, B). Similarly, the number of cells that are dividing (mitotic cells, arrowheads, Figure 4A) increased by 25% in *Wnt5a−/−* mice at E11.5 (Figure 4A, C). Interestingly, the increase of cells in S-phase and M-phase was maintained along the entire antero-posterior axis of the VM.

**Figure 5:**
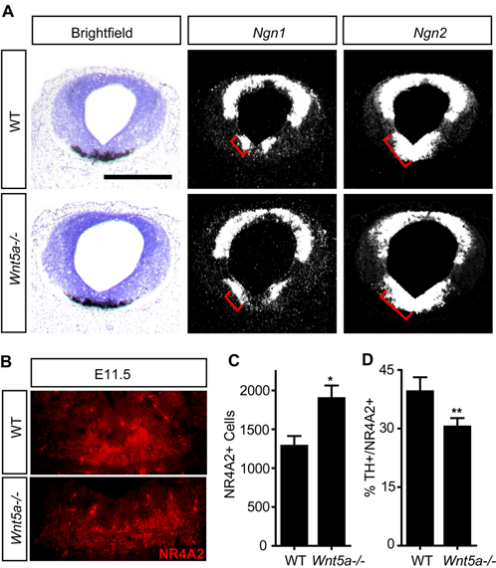
The ventral midbrain progenitor domains are expanded in *Wnt5a−/−* embryos. (A) Detection of *Th*, *Ngn1*, and *Ngn2* on representative serial coronal sections at the level of the midbrain of WT and *Wnt5a−/−* embryos at E11.5. Red brackets delimit the ventral *Ngn1* and *Ngn2* domain in the WT embryo, respectively. (Scale bar in A: 500 µm.). (B) Increase in NR4A2+ cells in the VM of *Wnt5a−/−* mice, which occupy a broader region laterally and dorsoventrally. (C) A 47% increase in the number of NR4A2+ cells was detected in the *Wnt5a−/−* mice at E12.5 (unpaired t-test, p = 0.044, WT N = 3, *Wnt5a−/−* N = 4). (D) Analysis of the proportion of NR4A2+ cells differentiating into TH+ DA neurons revealed that the differentiation of NR4A2+ precursors into TH+ cells was impaired in the *Wnt5a−/−* mice at E12.5 (paired t-test, p = 0.0098, N = 3).

Neurogenesis in the VM is controlled by a code of homeodomain and proneural bHLH transcriptional regulators [44]. Expression of the members of the bHLH superfamily *Ngn1*, *Ngn2* and *Mash1* can be subdivided into three distinct domains within the VZ of the mouse VM [45]. *Ngn2* is essential for proper DA neurogenesis, and *Mash1* is required for the generation of VM GABAergic neurons [45]–[47]. The expression of *Ngn2* (and the low levels of *Mash1*) in the midbrain FP therefore define the DA progenitor domain, whereas the high expression levels of *Ngn2* and *Ngn1* in the adjacent basal plate (BP) demarcates the oculomotor (OM) and red nucleus (RN) progenitor domains [45], [46], [48]. In the *Wnt5a−/−* embryos, the *Ngn1*-positive domain within the BP and the *Ngn2*-positive domain comprising the FP and BP were broadened in the mutant midbrain at E11.5 (Figure 5A), whereas the expression domains of these two proneural factors remained unchanged in the dorsal midbrain. We next examined whether the increase in cell division and broadened neurogenic domain had generated more postmitotic cells, and found a 47% increase in the number of NR4A2+ postmitotic cells compared to control at E12.5, suggesting that the neurogenic process is indeed enhanced in the *Wnt5a−/−* mice (Figure 5B, C). Furthermore, the 47% increase in NR4A2+ cells is consistent with a 45% increase in proliferating cells. However, a 25% excess of TH+ at E14.5 indicates that not all of the excess NR4A2+ cells differentiate into Th+ neurons at this point. Moreover, we found that the domain occupied by NR4A2+ cells was wider in *Wnt5a−/−* than in wild type mice (Figure 5B). An increase in the amount of NR4A2 mRNA was also detected at E12.5 by QPCR (Figure S2C).

We have previously reported that one of the functions of Wnt5a is to promote the differentiation of NR4A2+ DA precursors into DA neurons *in vitro*. This has been shown in diverse *in vitro* preparations, including rat E14.5 VM primary cultures [32], VM neurospheres derived from E10.5 mice or E12.5 rat [49] and mouse ES cells (unpublished observation). In order to investigate whether Wnt5a also promotes this differentiation step *in vivo*, we examined the proportion of TH+ cells out of NR4A2+ cells in the VM of *Wnt5a−/−* mice at E12.5. In agreement with our previous *in vitro* results, we found that the proportion of NR4A2+ cells differentiating into Th+ cells was reduced by 23% at this stage (Figure 5C). However since the number of TH+ cells increased at E14.5, and subsequently normalized, our results suggest that differentiation is rescued by an alternative mechanism. We thus conclude that Wnt5a is only partially and transiently required for the differentiation of NR4A2+ precursors into DA neurons *in vivo*.

### Mediolateral/PCP and apicobasal morphogenetic defects in the VM of *Wnt5a−/−* mice

**Figure 6:**
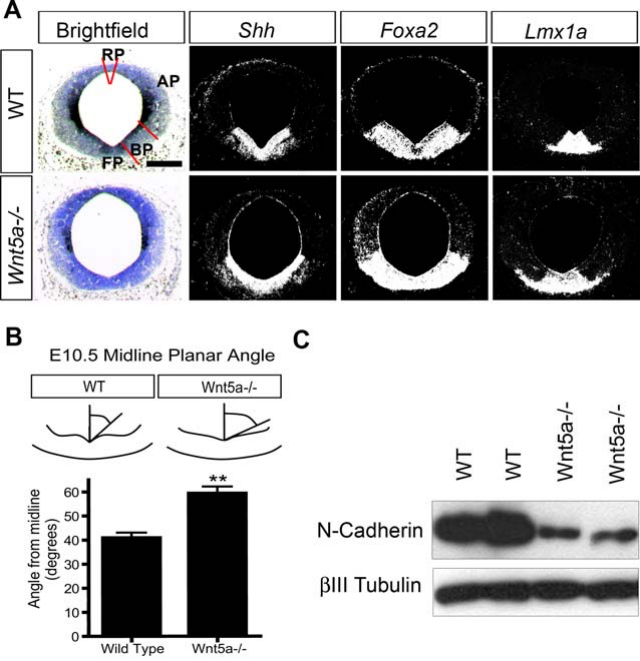
Lateral expansion of *Sonic hedgehog* expression and flattening of midbrain ventricle, associated with loss of N-cadherin in *Wnt5a−/−* embryos. (A) *Shh*, *Foxa2* and *Lmx1a* are laterally expanded by E11.5 in *Wnt5a−/−* mice. The invagination at the medial hinge-point is markedly reduced/flattened in the mutants. Red bars delimit the alar plate (AP), basal plate (BP), floor plate (FP) and roof plate (RP). Scale bar 250 µm. (B) The flattened VM invagination was quantified by measuring the angle formed between the midline and the ventricular wall at several levels throughout the VM of E10.5 mice, when this morphological change first became obvious. A significant difference was found in the *Wnt5a−/−* mice forming a U-shaped ventricle, with an angle of 59.4°±2.8° from the midline as compared to 49.1°±4.3° in WT mice. (C) Western blot of E9.5 whole brain of WT and *Wnt5a−/−* mice revealed a marked reduction of N-cadherin in *Wnt5a−/−* mice.

Several of the defects described in previous sections are reminiscent of PCP developmental defects, including the accumulation of BrdU+ cells in the midline, the lateral expansion of the Ki67+, Ngn2+, NR4A2+ and TH+ domains in the VM as well as a flattening of the midbrain ventricle (Figures 3, 4, 5). Previous reports of animals lacking PCP components have shown that the invagination of the VZ in the ventral midline is flattened and that the Shh domain, that defines the FP and BP in the midbrain, is broadened [50]–[52]. In the *Wnt5a−/−* mice, a lateral expansion of the *Shh* and *Foxa2* expression domains was first detected at E11.5 (Figure 6A, Figure S2D,E). This was associated with a lateral expansion of the *Lmx1a+* DA progenitor domain (Figure 6A). Expression of the Shh-target genes (*Ptch1* and *Gli1*) was not expanded in the midbrain AP (Figure S3). The expression of class I (*Dbx1*) and class II (*Nkx2-2*, *Nkx6-1*) genes was not changed in the midbrain of the *Wnt5a−/−* embryos compared to WT although the aberrant morphology of the *Wnt5a−/−* midbrain noted previously led to a wider separation of the *Nkx6-1*-positive domains in the BP of the mutant midbrain (Figure S3 and data not shown). To characterize this defect in more detail, the analysis of the angle formed between the invagination of the ventricular epithelia and the midline in the VM revealed that this angle appeared greater in *Wnt5a−/−* mice (59.4°±2.8°), than in WT mice (49.1°±4.3°) (Figure 6B). This resulted in the midbrain ventricle adopting a “U”-shape in the *Wnt5a−/−* mice instead of the typical “V”-shape in controls (Figure 6A, B). A similar phenotype, including a broadened Shh domain, has been observed in the neural tube of the PCP mutants Scribble and Vangl2 [50]–[52].

It has previously been shown that cadherins regulate intercellular adhesion in neural progenitors and that a disruption of the complex formed with α- and β-catenin leads to increased Shh signaling and proliferation [53]. We therefore examined the levels of N-cadherin in *Wnt5a−/−* mice by Western blot and found a reduction at E9.5, prior to the expansion of the FP (Figure 6C).

**Figure 7:**
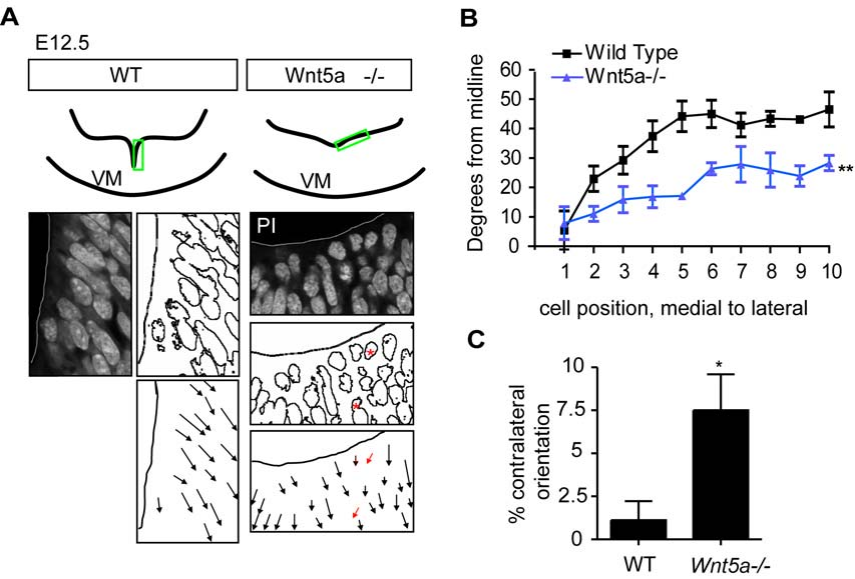
Apical-basal polarity/cell orientation are affected in *Wnt5a−/−* mice. (A) At E12.5, propidium iodide staining on coronal sections through the VM revealed that cell nuclei in the neuroepithelium were rounded and their orientation was more variable with some cells pointing contralaterally (red asterisks/arrows). (B) The orientation of each cell nucleus was plotted versus its distance from the ventral midline. The angle between the nucleus and the midline was measured from cell 1 (the most medial) to cell 10 (the most lateral). Cell nuclei in *Wnt5a−/−* mice are oriented more ventrally compared to the more lateral orientation of cells in WT mice (two way-ANOVA for genotype and level, p = 0.0029, N = 3). (C) The frequency of cell nuclei oriented towards the contralateral ventral side (red arrows in A) was significantly increased in *Wnt5a−/−* mice (paired t-test, p = 0.0198, N = 3, 10 nuclei at 3 levels/animal).

Moreover, since apico-basal polarity depends on PCP and adhesion, we examined apico-basal polarity of cells in the ventricular zone. Interestingly, the change in the general morphology of the VM neuroepithelia was accompanied by an alteration in the orientation of the cells, as shown by the non-uniform orientation of propidium iodide stained nuclei in the *Wnt5a−/−* mice, compared to WT at E12.5 (Figure 7A). To assess this quantitatively, the angle formed by the longest axis of each nuclei with the ventral midline was measured for 10 cells (starting from the midline and counting laterally) at different anteroposterior levels of the VM. This angle changed from very acute to less acute as the cells were positioned further away from the midline in the wild type. This angle reached almost 50° in wild type, but did not exceed 30° degrees in *Wnt5a−/−* mice (Figure 7B). These results reflect the fact that, in *Wnt5a−/−* mice, the nuclei of apical FP cells are oriented ventrally, while the nuclei of control mice are oriented more ventro-laterally (Figure 7A, B). Furthermore, when the frequency of nuclei aberrantly oriented towards the contralateral side was examined, *Wnt5a−/−* mice showed a 7-fold increase compared to WT (Figure 7A, C). Note that while cells in the midline of both WT and *Wnt5a−/−* mice exhibited nuclei oriented contralaterally, only *Wnt5a−/−* mice showed nuclei with contralateral orientation in lateral positions of the FP (Figure 7A).

### Several neuronal populations in the VM are redistributed following the PCP defect

**Figure 8:**
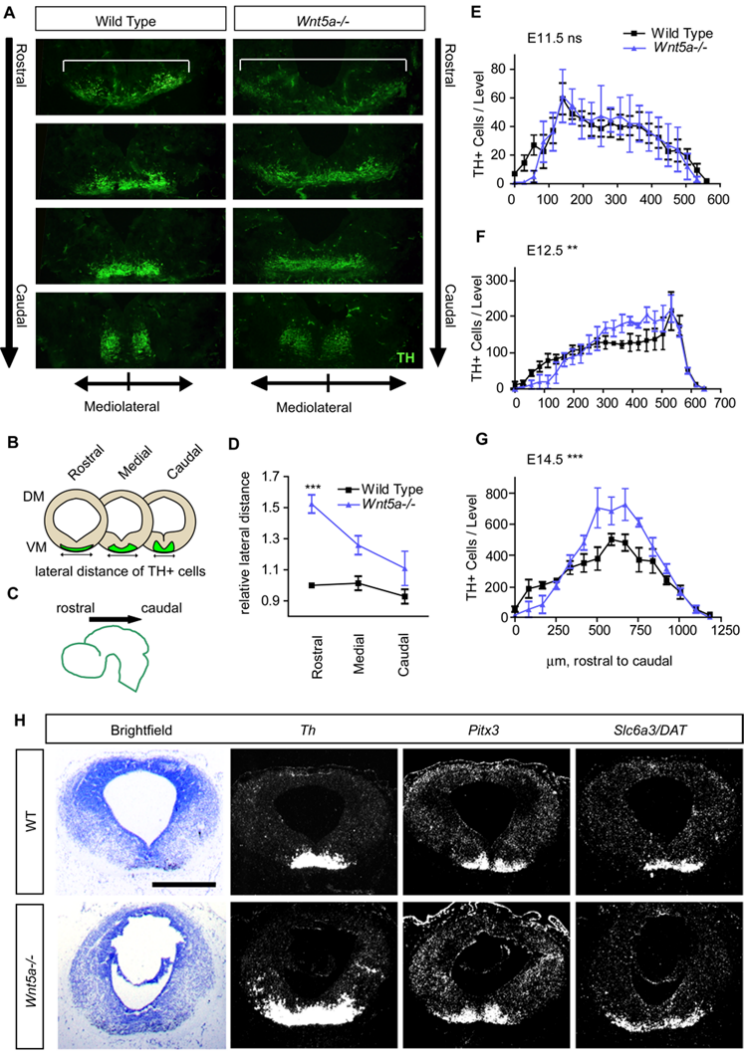
The distribution of TH+ cells in the rostrocaudal and mediolateral axis is altered in *Wnt5a−/−* mice. (A) At E12.5, there is a redistribution of TH+ cells in the VM, both in the rostrocaudal and mediolateral axis. (B) Three representative levels throughout the VM were measured for lateral spread of TH+ cells: rostral, medial and caudal. ImageJ was used to measure the distance between the two TH+ cells furthest apart in (D). (C) TH+ cells were quantified at regular intervals from rostral to caudal midbrain in (E–F). (D) TH+ cells are distributed much more broadly in anterior midbrain of E12.5 *Wnt5a−/−* mice compared to WT (ANOVA, N = 3, p<0.001). (ANOVA with Bonferroni's post test, Rostral WT vs Rostral *Wnt5a−/−* p<0.001). (E–F) Rostrocaudal distribution of TH+ cells. (E) At E11.5 no difference could be seen between the distribution of TH+ cells in the anteroposterior axis of WT or *Wnt5a−/−* mice. However, at E12.5 (F) and E14.5 (G) an altered distribution with a decrease in the number of TH+ cells in anterior levels and an increase in medial levels was seen (Two way ANOVA, for level and genotype, P = 0.0016 at E12.5, P<0.0001 at E14.5, N = 4). (H) The lateral expansion of TH+ cells at E12.5 was confirmed by *in situ hybridization* for *Th*, *Pitx3* and *DAT*, two other markers of maturing dopaminergic neurons.

A more detailed analysis of the distribution of A9–A10 DA neurons, in the very same animals that did not show any change in total DA cell number at E12.5, revealed changes in the mediolateral, rostro-caudal, and dorsoventral axis (Figure 8A). Sections at regular intervals throughout the A9–A10 nucleus were examined. The three levels analyzed in WT and *Wnt5a−/−* mice (rostral, intermediate and caudal) are shown at E12.5 in (Figure S4). In the mediolateral axis, TH+ cells extend more laterally in the *Wnt5a−/−* mice than in WT mice, especially in the rostral portion of the A9–A10 nuclei (Figure 8A–D). This phenotype persisted until E17.5, and followed the earlier morphological defects in the distribution of progenitor (*Shh*, *Foxa2*, *Lmx1a*, *Ngn2*) and precursor (NR4A2) markers, all of which were expanded already at E11.5.

In the rostro-caudal axis, the distribution of TH+ cells was unchanged at E11.5 (Figure 8E). However, at E12.5 and E14.5, the A9–A10 nuclei were shortened in *Wnt5a−/−* mice, seemingly at the expense of the most anterior portion of the nuclei (Figure 8F, G). Finally, by E17.5 the distribution of VM DA neurons in the anteroposterior axis tended to normalize but remained broad in the dorsoventral axis (Figure 3B). This shortening of the anterior neural tube at E12.5 was also apparent when the length of the *En1* expression domain was measured in sagittal sections through the VM of *Wnt5a−/−* mice, and compared to WT (390 µm compared to 540 µm, Figure S5).

To distinguish between a deregulation of TH and a true misplacement of the DA neurons, we examined the spatio-temporal expression pattern of other DA neuron markers such as *Pitx3* and *Slc6a3/Dat*. Both marker genes were expressed within the marginal zone (MZ) of the *Wnt5a−/−* ventral midbrain at E12.5 (Figure 8H). A mediolateral expansion of the *Th*, *Pitx3* and *Slc6a3*/*DAT* expression domains was detected in the *Wnt5a−/−* mice, confirming the redistribution of DA neurons within the VM (Figure 8H).

Since Wnt5a regulates PCP in several structures and the *Shh*, *Foxa2* and *Ngn1* domains were also broadened in the BP, we examined whether deletion of *Wnt5a* altered the distribution of other mature ventral neuronal populations in coronal midbrain sections at E18.5, when no difference in number of DA neurons was detected (data not shown). Interestingly, the area occupied by *Th−*, *Islet1*− or *Brn3a*− expressing cells was increased in *Wnt5a−/−* mutants (30–40%, Table 1). These results indicate that while the absolute numbers of midbrain neuron populations such as DA neurons are unchanged, the cells are re-distributed and positioned in a laterally and dorsoventrally enlarged domain in the *Wnt5a−/−* VM (Table 1 and Figure 3).

**Table 1:** The area occupied by the *Islet1+*, *Brn3a+* or *Th+* domain in coronal sections is 30–40% greater in *Wnt5a−/−* mice at E18.5.

Genotype	*Isl1* ^+^ domain (µm^3^×10^5^) n = 4	*Brn3a* ^+^ domain (µm^3^×10^5^) n = 3	*Th* ^+^ domain (µm^3^×10^5^) n = 3
**Wildtype**	62,4±9,79 s.e.m.	347,2±20,8 s.e.m.	805,87±13,11 s.e.m.
***Wnt5a−/−***	89,3±4,05 s.e.m.	443,2±18,41 s.e.m.	1140,27±9,34 s.e.m.
**Student's T-test**	0.044195531 *	0.025901993 *	3.17277E-05 ***

The more ventral *Th+* population shows the greatest difference.

## Discussion

The impact of deletions of Wnt/PCP signaling components [2], [29], including ligands such as Wnt5a [4], have been studied in very specific structures that have become standard Wnt/PCP functional assays. These include studying the orientation of bristles on the *Drosophila* wing, and in mammals the orientation of hair cells in the inner ear, convergent extension movements during embryo elongation, or neural tube closure. However, Wnt/PCP signaling components and ligands are expressed in very diverse tissues and at multiple developmental stages, where no previous PCP phenotypes have been described. In our study we investigated the function of Wnt5a in one such structure and developmental time, the VM during neurogenesis. We report that Wnt5a regulates VM morphogenesis, limits DA progenitor proliferation and enhances DA precursor differentiation.

### Regulation of DA precursor differentiation by Wnt5a

Despite the clear effects of Wnt5a on DA differentiation in both gain and loss of function experiments *in vitro* [32], [54], *Wnt5a−/−* mice exhibited only a mild and transient DA differentiation phenotype *in vivo*. Moreover, in addition to a decrease in the proportion of NR4A2+ precursors differentiating into TH+ DA neurons (25% decrease in the Wnt5a mutants compared to WT), we found that NR4A2+ DA precursors were generated in excess by E12.5 (47% in the Wnt5a mutants compared to 39% in WT). These results suggest that both progenitor proliferation and the differentiation of NR4A2+ precursors were affected in the *Wnt5a* mutants. These alterations were transient and were not detected at E14.5, when the number of TH+ cells increased by 25%, then returned to control level at E17.5 and E18.5. Thus, our results show that Wnt5a is only transiently required for the differentiation of endogenous midbrain NR4A2+ precursors *in vivo*, and is sufficient for their differentiation *in vitro*, via Rac1 activation. Interestingly, the surprising alteration in the kinetics of DA neurogenesis in the *Wnt5a−/−* mice suggests that other phenomena such as increased neurogenesis (increased number of DA postmitotic precursors) and a compensatory mechanism at E14.5, may prevent a stronger DA differentiation phenotype. Indeed, several non-canonical Wnts are expressed in the VM [37], and a functional redundancy between wnt5 and wnt11 has been described in double zebrafish *pipetail* (*ppt*)/*wnt5* and *silberblick* (*slb*)/*wnt11* mutants [30], suggesting that another non-canonical Wnt may compensate for the loss of Wnt5a.

### A role for Wnt5a in midbrain morphogenesis

The involvement of non-canonical Wnt-signaling in CE movements and neural tube morphogenesis has been widely documented [29], [55], [56]. Moreover, Wnt5a has recently been clearly implicated in the regulation of CE movements and PCP during cochlear development and neurulation in the mouse [4]. Interestingly, the mesencephalon undergoes unique morphogenic movements to form the cephalic flexure proper, a process that is coupled to CE movements in the ventral domain and outgrowth in the dorsal domain of the mesencephalon [57]. Moreover, *Wnt5a* is expressed exclusively in the VM and not dorsal midbrain throughout mouse embryonic development. Our analysis of the *Wnt5a−/−* mice has revealed a large variety of subtle but clear alterations in the morphogenesis and the cytoarchitecture of the VM. Overall the VM was broader and shorter as demonstrated by: a lateral expansion of the *Shh*-expressing FP/BP at E11.5, accompanied by broader lateral expression of *Foxa2*, *Lmx1a*, NR4A2, Th, *Pitx3* and *DAT*. Other markers examined were also more laterally placed such as *Wnt1* and *Nkx6.1*. The shortened midbrain phenotype was manifested in the number of levels with TH+ cells and their distribution, and suggested by a reduced length of *En1* expression in sagittal sections. In coronal sections, the mesencephalon did not acquire its distinct heartlike morphology and the ventricle in the ventral midline adopted a wide “U” shape instead of the typical “V” shape at E10.5. Thus, our findings suggest diminished CE and an involvement of Wnt/PCP signaling in the VM, as described for other structures [4], [22], [24], [28], [58].

Interestingly, we also found a change in the apico-basal orientation of midbrain VZ cells in *Wnt5a−/−* mice compared to WT. The axis of individual WT FP apical ventricular cells and the midline formed an average 36° angle and did not cross the midline ventrally. However, cells in the *Wnt5a−/−* mice formed an average 20° angle with the midline and in some instances individual cells showed a negative angle (i.e. their axis pointed contralaterally). A possible interpretation of these results is that the increase in proliferation allows nuclei to be oriented aberrantly in *Wnt5a−/−* mice during mitosis. However, misorientation was never seen in WT mice in lateral positions, regardless of cell cycle phase, but was seen in *Wnt5a−/−* mice. This could also reflect a consequence of the flattened VM morphology, allowing greater deviations towards the contralateral side. A more intriguing possibility is that the alteration in PCP directly regulates or causes secondary changes in the geometry of the cells and in apicobasal polarity. PCP and apicobasal polarity require cell attachment, a process that both pathways also regulate [59], [60]. In line with this, we found a decrease in the levels of N-cadherin in the *Wnt5a−/−* mice at E9.5, which could contribute to decreased attachment and altered polarity. These results suggest PCP and apico-basal polarity may be coordinately regulated in certain structures by adhesion proteins.

Triple *slb*;*ppt*;*wnt4*-morphant zebrafish embryos phenocopy the neurulation defect of *trilobite* (*tri*)/*Van Gogh-like 2* (*Vangl2*) PCP mutants [61]. Interestingly, the defects in the *Wnt5a−/−* VM resemble the *tri* neurulation defects with an increase in proliferation, a broadening of the *Shh* domain/expansion of the FP, and an ectopic accumulation of neural progenitors within the midline [61]. These findings strongly suggest that Wnt5a also regulates cell intercalation after cell division and VM morphogenesis. Our analysis of the VM phenotype of *Wnt5a−/−* mice suggests a model in which Wnt5a regulates morphogenesis by a mechanism involving cell adhesion and altered PCP signaling. This is accompanied by an alteration in apico-basal polarity, and a broader distribution of VM cell populations, but no alteration in the final number of neurons, all of which is compatible with a PCP phenotype.

### Proliferation: a link between differentiation and morphogenesis

Our results indicate that the loss of Wnt5a directly or indirectly regulates cell division in VZ progenitors. Indeed, we found that deletion of *Wnt5a* increased the number of both BrdU+ and Ki67+ cells in the apical VZ, and the number of NR4A2+ postmitotic cells in the IZ, which suggested a regulation of both proliferative and neurogenic divisions. Interestingly, Wnt/PCP signaling has been previously reported to regulate oriented cell division during gastrulation, allowing axis elongation [61], [62], and to regulate asymmetric cell division [63], which is the predominant mode of division used by VM progenitors during DA neurogenesis. Another mechanism that could also result in the regulation of proliferation and neurogenesis is cell adhesion. Our results indicate that Wnt5a may regulate cell adhesion in the neuroepithelia by regulating the levels of N-cadherin as early as E9.5. Interestingly, it has been described that a reduction in α-catenin binding to N-cadherin leads to decreased cell adhesion, increased Shh signaling and proliferation [53]. We therefore suggest that the increase in Shh signaling (first detected at E11.5 by the regulation of *Foxa2* and *Lmx1a*), may be induced by a decrease in the levels of N-cadherin protein and cell adhesion at earlier stages, when no other phenotype is apparent. In agreement with a role for Shh in regulating proliferation, we found an increase in the number of proliferating apical VZ progenitors and a lateral expansion of the *Shh*, *Foxa2* and *Lmx1a* domains in the *Wnt5a−/−* mice. Thus, taken together, these data suggest that Wnt5a can affect both the extent and the type of cell division in VZ progenitor cells by different mechanisms.

In sum, our results show that CE defects and lowered cadherin expression were the first detected phenotype at E9.5 in the *Wnt5a−/−* mice. This was followed by an alteration in morphogenesis and DA differentiation, and at E12.5 by defects in cell polarization and adhesion, a shortening and a broadening of the A9–A10 nucleus and other ventral domains of the mesencephalon. We suggest that the broadening of the Shh domain may lead to an increase in Shh-signaling, increased proliferation in the FP/BP, accumulation of NR4A2+ precursors and a transient expansion of the Th+ cell population at E14.5. These defects were most pronounced in the FP, thus affecting the morphology and development of cells in the DA lineage in the *Wnt5a−/−* mice.

In conclusion, we show for the first time that, in the VM, Wnt5a regulates CE movements required for axial elongation, polar growth and morphogenesis as well as proliferation, neurogenesis, some aspects of differentiation, and the actual positioning of neurons in the VM.

## Materials and Methods

### Animals, Immunohistochemistry and in situ Hybridization (ISH)

*Wnt5a^tm1Amc^* (referred to as *Wnt5a−/−* in this article) transgenic mice [38] and CD1 mice (Charles River) were housed, bred and treated in accordance with the approval of the local ethics committee (Stockholms Norra Djurförsöketiska Nämnd). *Wnt5a+/−* mice were kept on a C57BL/6 background. In all experiments, the *Wnt5a−/−* embryos were compared to their wild-type (*Wnt5a+/+* and *Wnt5a+/−*, heterozygotes have wild-type phenotype) littermates, n≥4 for each genotype, if not otherwise stated in the text. Mice of the relevant genotype were mated overnight and noon of day of plug was taken as E0.5. Embryos were dissected in ice-cold PBS, fixed in 4%PFA 4 hrs to overnight, cryoprotected in 20% sucrose and frozen in OCT compound on dry ice. Serial coronal 7, 14 or 16 µm sections of the brain were obtained on a cryostat. Immunohistochemistry (IHC) and in situ hybridization (ISH) were carried out as previously described [32], [64]. IHC and ISH for *Wnt5a* were visualized with a Zeiss HBO100 microscope; images were collected with a C4742-95 Hamamatsu camera and processed with OpenLab software and/or ImageJ.

### Antibodies

Rabbit anti-Nurr1 (NR4A2) (1∶200, Santa Cruz Biotech.), rabbit anti-TH (1∶500, Pel-Freeze), mouse anti-Ki67 (1∶800, Abcam), anti-BrdU, Cy2-, Cy3- or Rhodamine-coupled secondary antibodies (1∶250, Jackson ImmunoResearch, West Grove, PA/USA), or biotin-conjugated goat anti-rabbit IgG (1∶300) were used. Some sections were counterstained with 4′, 6-Diamino-2-phenylindole (DAPI) (500 ng/ml, Sigma, Missouri/USA), or propidium iodide (Invitrogen). Anti-active-β-catenin (1∶500, Clone 8E7 from Upstate Biotechnology/Millipore), anti Rac1 (1∶1000, Upstate), anti RhoA (1∶1000, sc418 Santa Cruz), anti CDC42 (1∶1000, Trans Lab) and anti-β-actin (1∶5000, Abcam) were used for Western blot.

### Radioactive in situ hybridization

Paraffin sections (8 µm) of mouse embryos (E11.5/E12.5) or brains (E18.5, P56) were processed for radioactive in situ hybridization as previously described [65]. The probes used were as follows: *Wnt5a* [38], *Shh*, *En1*, *Wnt1* [66], *Th*, *Pitx3*, *Slc6a3*/*Dat* [65], *Foxa2* (bp 743–1314, Acc. Nr. NM_010446), *Dbx1*, *Isl1*, *Pou4f1*, *Nkx2-2*, *Nkx6-1* [67], *Ngn1*, *Ngn2* [68], *Lmx1a* (bp 412–1211, Acc. Nr. NM_033652) *Ptch1* [69] and *Gli1* [70]. Images were taken using darkfield optics on a stereo microscope Stemi SV6, AxioCam MRc camera and Axiovision 4.6 software (Zeiss, Jena/Germany).

### BrdU treatments

Pregnant females were injected intra-peritoneally with 5-bromo-2-deoxyuridine (BrdU, 10 µg/g body weight) two hours before they were sacrificed and processed as described [71]. The number of BrdU-positive cells in a square area (2500 µm2) was counted on at least four serial sections from the midbrain of stage-matched littermates (n = 4) using the Neurolucida 6 software (MBF Bioscience, Williston, VT/USA).

### *TH+* Cell Counts and Distribution

For counts of total number or distribution of TH+ cells in the ventral midbrain, alternate 14 µm sections from coronal series of the entire A9–A10 population were counted and plotted versus position at E11.5 and E12.5 for distribution analysis. At E14.5 and E17.5, every sixth section was counted and plotted in the distribution analysis. The lateral spread of TH+ cells was measured in coronal sections through the VM, in three consecutive levels, where level 1 corresponds to the rostral midbrain-P1 boundary, level 2 corresponds to the intermediate midbrain and level 3 corresponds to the caudal midbrain (see Figure S 5). Pictures were taken with identical acquisition settings and magnification and the lateral spread was measured by drawing a horizontal line between the outermost TH+ cells. This distance was measured in pixels using ImageJ and then normalized to littermate controls.

The volumes of the *Th*−, *Brn3a*− and *Isl1*-expression domains at E18.5 were measured by the Cavalieri method using the Stereo Investigator 5.05.4 software (MBF Bioscience) as described [65]. Cell numbers were averaged for each genotype and subjected to a Student's t-test for the estimation of statistical significance.

### Calculation of slope of ventricular invagination and analysis of nuclei orientation

E10.5 and E12.5 mice were stained with Hoechst or propidium iodide respectively. The slope of the wall of the VM at E10.5 was measured by drawing a cross bisecting the FP, where the origin coincided with the ventricular invagination. A tangent to the ventricular wall was drawn and the angle between the tangent and the midline was measured in 4 embryos of each genotype. To measure the nucleus orientation, confocal pictures of serial sections of E12.5 midbrains were taken with an optical slice of 1 µm. Ten nuclei along the ventricle were analyzed per image from the midline outwards, 3 images per mouse (rostro-caudally distributed), and 3 mice per genotype were analyzed. The nucleus was outlined in Image J and the angle of the longest axis was measured compared to the vertical parallel to the midline. This gave an angle that was plotted against its position from the midline (nucleus number position). Certain nuclei were abnormally oriented towards the contralateral side, these gave a negative angle. The number of nuclei with abnormal orientation (negative angle), was compared to the total number of cells and this ratio was used for analysis of contralateral orientation.

### Primary ventral mesencephalic cultures and immunocytochemistry

Ventral midbrains of E11.5 CD1 mice were dissected out in ice-cold PBS supplemented with 0.2%glucose, mechanically dissociated in serum-free N2 through flame-narrowed Pasteur pipettes and plated at a final density of 125,000 cells/well in poly-D-lysine-coated 48-well plates as described previously [32]. Cultures were treated with recombinant mouse Wnt5a (200 ng/ml, RnD), 0.05% CHAPS (Wnt5a control, Sigma), and/or 10 µM Rac1 inhibitor (NSC 23766, Calbiochem). Treatment of cultures was initiated 1 hour after plating and cultures were incubated for 3 days in N2 at 37°C in 5%CO_2_. Cells were then fixed for 15 minutes with 4% paraformaldehyde, washed in PBS, and used for immunocytochemical analysis.

### Real-time Quantitative PCR

cDNA was generated as described previously [32]. In brief, RNA from E8.5-P0 VMs of CD1 (for developmental stages) or wildtype and *Wnt5a−/−* mice was extracted using RNeasy Mini Kit (Qiagen). 1 µg of RNA was reverse-transcribed using SuperScript II Reverse Transcriptase (Invitrogen) and random primers (Invitrogen). Quantitative PCR and the primers used in this study have been described previously [32] with the exception of NR4A2 primers; Forward: 5′CAGCTCCGATTTCTTA ACTCCAG3′ and Reverse: 5′GGTGAGGTCCATGCTAAACTTGA3′.

### Activated GTPase assay

Immunoblotting and sample preparation were performed as described [17]. SN4741cells were cultured as described previously [17]. Cells were serum starved overnight and stimulated with recombinant Wnt5a (200 ng/ml, R&D) for 2 hours. Cells were washed with ice-cold PBS and lysed in GTPase lysis buffer (10 mM Tris-HCl, pH = 7.5; 110 mM NaCl; 1 mM EDTA; 10 mM MgCl_2_; /well, 1% Triton – ×100; 0.1% SDS, 1 mM DTT, protease inhibitor cocktail (Roche)) for 5 minutes at 4°C. Cell lysates were cleared by centrifugation at 14 000 rpm/4°C/5 min and incubated for 15 min/4°C on a rotator with 25 µl of GST-PAK-CRIB beads (25% slurry) for Rac1-gtp pulldown, GST-Rhotekin for RhoA-gtp pulldown and GST-WASP for cdc42-gtp pulldown. Beads were subsequently washed 3 times with 0.5 ml GTPase lysis buffer (without 0.1% SDS), mixed with 2× Laemmli buffer and analyzed on SDS-PAGE for the amount of activated RhoA/Rac1/cdc42. 5% of total cell lysate was used as input control.

### Statistics

Data were analysed using Graphpad Prism version 4.01 for Windows, GraphPad Software, San Diego California USA, www.graphpad.com. For analyses comparing WT and *Wnt5a−/−* mice, t-tests were used. A paired t-test was used if 1WT/Wnt5a littermate pair was used from individual litters. If several pairs were taken from the same litter then an unpaired t-test was used. At least 3 litters were used for each analysis. Each graph value is the mean±SEM. For analyses of distribution of Rac inhibition of dopaminergic neuron differentiation and for dopaminergic neuron distribution *in vivo*, One-way and Two-way ANOVA were used, with Bonferroni's post-hoc test.

## Supporting Information

Figure S1 Canonical Wnt signaling is unaffected by loss of *Wnt5a*. (A) *In situ hybridization* at E12.5 for *Wnt1* shows wider spacing between the two ventral stripes of *Wnt1* expression in the *Wnt5a−/−* mice (red brackets), but no enlargement of the *Wnt1* domain (Scale bar is 500 µm). (B) QPCR for *Wnt1* shows no difference in levels of *Wnt1* in the VM of *Wnt5a−/−* mice at E12.5. (C) Western blot for active (dephosphorylated) β-catenin shows little difference at E12.5 in the *Wnt5a−/−* VM.
(4.57 MB TIF)

Figure S2 QPCR of E12.5 WT and *Wnt5a−/−* VM. No difference in *Th* (A) and *Pitx3* (B) mRNA levels. Increase in the amount of *Nr4a2* mRNA (C). Small or no increase in *Shh* mRNA (D) and a greater difference in *Foxa2* levels (E).
(0.71 MB TIF)

Figure S3 No change in dorsoventral patterning of the *Wnt5a−/−* midbrain. Expression of *Shh*-target genes *Ptch1* and *Gli1* was not altered in *Wnt5a−/−* mice. The expression of class I (*Dbx1*) and class II (*Nkx6-1*) genes was not changed in the midbrain of the *Wnt5a−/−* embryos compared to WT.
(5.40 MB TIF)

Figure S4 Anteroposterior levels used to analyze the lateral distribution of TH+ cells. Levels 1, 2 and 3 corresponding to rostral, intermediate and caudal levels are depicted on sagittal sections of WT and *Wnt5a−/−* mice, probed for *Th*.
(3.81 MB TIF)

Figure S5 The *En1*-expressing domain is shortened in *Wnt5a−/−* VM (A) Bright field and dark field images of the midbrain cephalic flexure hybridized for *Engrailed1* (*En1*) show a shorter anteroposterior extension of *En1* in the *Wnt5a−/−* midbrain. Red line shows the length measured from isthmus to anterior-most *En1* expression. (B) Quantification of *En1* expression length shows a shorter domain in *Wnt5a−/−* embryos (paired t-test p = 0.0203, WT N = 3, *Wnt5a−/−* N = 2).
(7.93 MB TIF)

## References

[1] Clevers H (2006). Wnt/beta-catenin signaling in development and disease.. Cell.

[2] Seifert JR, Mlodzik M (2007). Frizzled/PCP signalling: a conserved mechanism regulating cell polarity and directed motility.. Nat Rev Genet.

[3] Mikels AJ, Nusse R (2006). Purified Wnt5a protein activates or inhibits beta-catenin-TCF signaling depending on receptor context.. PLoS Biol.

[4] Qian D, Jones C, Rzadzinska A, Mark S, Zhang X (2007). Wnt5a functions in planar cell polarity regulation in mice.. Dev Biol.

[5] Witze ES, Litman ES, Argast GM, Moon RT, Ahn NG (2008). Wnt5a control of cell polarity and directional movement by polarized redistribution of adhesion receptors.. Science.

[6] Wodarz A, Nusse R (1998). Mechanisms of Wnt signaling in development.. Annu Rev Cell Dev Biol.

[7] Vinson CR, Adler PN (1987). Directional non-cell autonomy and the transmission of polarity information by the frizzled gene of Drosophila.. Nature.

[8] Djiane A, Riou J, Umbhauer M, Boucaut J, Shi D (2000). Role of frizzled 7 in the regulation of convergent extension movements during gastrulation in Xenopus laevis.. Development.

[9] Wang Y, Guo N, Nathans J (2006). The role of Frizzled3 and Frizzled6 in neural tube closure and in the planar polarity of inner-ear sensory hair cells.. J Neurosci.

[10] Montcouquiol M, Rachel RA, Lanford PJ, Copeland NG, Jenkins NA (2003). Identification of Vangl2 and Scrb1 as planar polarity genes in mammals.. Nature.

[11] Wolff T, Rubin GM (1998). Strabismus, a novel gene that regulates tissue polarity and cell fate decisions in Drosophila.. Development.

[12] Curtin JA, Quint E, Tsipouri V, Arkell RM, Cattanach B (2003). Mutation of Celsr1 disrupts planar polarity of inner ear hair cells and causes severe neural tube defects in the mouse.. Curr Biol.

[13] Das G, Reynolds-Kenneally J, Mlodzik M (2002). The atypical cadherin Flamingo links Frizzled and Notch signaling in planar polarity establishment in the Drosophila eye.. Dev Cell.

[14] Wallingford JB, Rowning BA, Vogeli KM, Rothbacher U, Fraser SE (2000). Dishevelled controls cell polarity during Xenopus gastrulation.. Nature.

[15] Sussman DJ, Klingensmith J, Salinas P, Adams PS, Nusse R (1994). Isolation and characterization of a mouse homolog of the Drosophila segment polarity gene dishevelled.. Dev Biol.

[16] Strutt H, Price MA, Strutt D (2006). Planar polarity is positively regulated by casein kinase Iepsilon in Drosophila.. Curr Biol.

[17] Bryja V, Schulte G, Rawal N, Grahn A, Arenas E (2007). Wnt-5a induces Dishevelled phosphorylation and dopaminergic differentiation via a CK1-dependent mechanism.. J Cell Sci.

[18] Klein TJ, Jenny A, Djiane A, Mlodzik M (2006). CKIepsilon/discs overgrown promotes both Wnt-Fz/beta-catenin and Fz/PCP signaling in Drosophila.. Curr Biol.

[19] Fanto M, Weber U, Strutt DI, Mlodzik M (2000). Nuclear signaling by Rac and Rho GTPases is required in the establishment of epithelial planar polarity in the Drosophila eye.. Curr Biol.

[20] Habas R, Dawid IB, He X (2003). Coactivation of Rac and Rho by Wnt/Frizzled signaling is required for vertebrate gastrulation.. Genes Dev.

[21] Winter CG, Wang B, Ballew A, Royou A, Karess R (2001). Drosophila Rho-associated kinase (Drok) links Frizzled-mediated planar cell polarity signaling to the actin cytoskeleton.. Cell.

[22] Yamanaka H, Moriguchi T, Masuyama N, Kusakabe M, Hanafusa H (2002). JNK functions in the non-canonical Wnt pathway to regulate convergent extension movements in vertebrates.. EMBO Rep.

[23] Wilson PA, Oster G, Keller R (1989). Cell rearrangement and segmentation in Xenopus: direct observation of cultured explants.. Development.

[24] Wallingford JB, Fraser SE, Harland RM (2002). Convergent extension: the molecular control of polarized cell movement during embryonic development.. Dev Cell.

[25] Adler PN (2002). Planar signaling and morphogenesis in Drosophila.. Dev Cell.

[26] Dabdoub A, Donohue MJ, Brennan A, Wolf V, Montcouquiol M (2003). Wnt signaling mediates reorientation of outer hair cell stereociliary bundles in the mammalian cochlea.. Development.

[27] Wallingford JB, Harland RM (2002). Neural tube closure requires Dishevelled-dependent convergent extension of the midline.. Development.

[28] Ybot-Gonzalez P, Savery D, Gerrelli D, Signore M, Mitchell CE (2007). Convergent extension, planar-cell-polarity signalling and initiation of mouse neural tube closure.

[29] Wang Y, Nathans J (2007). Tissue/planar cell polarity in vertebrates: new insights and new questions.. Development.

[30] Kilian B, Mansukoski H, Barbosa FC, Ulrich F, Tada M (2003). The role of Ppt/Wnt5 in regulating cell shape and movement during zebrafish gastrulation.. Mech Dev.

[31] Ulrich F, Concha ML, Heid PJ, Voss E, Witzel S (2003). Slb/Wnt11 controls hypoblast cell migration and morphogenesis at the onset of zebrafish gastrulation.. Development.

[32] Castelo-Branco G, Wagner J, Rodriguez FJ, Kele J, Sousa K (2003). Differential regulation of midbrain dopaminergic neuron development by Wnt-1, Wnt-3a, and Wnt-5a.. Proc Natl Acad Sci U S A.

[33] Schulte G, Bryja V, Rawal N, Castelo-Branco G, Sousa KM (2005). Purified Wnt-5a increases differentiation of midbrain dopaminergic cells and dishevelled phosphorylation.. J Neurochem.

[34] Wallingford JB, Vogeli KM, Harland RM (2001). Regulation of convergent extension in Xenopus by Wnt5a and Frizzled-8 is independent of the canonical Wnt pathway.. Int J Dev Biol.

[35] Rawal N, Castelo-Branco G, Sousa KM, Kele J, Kobayashi K (2006). Dynamic temporal and cell type-specific expression of Wnt signaling components in the developing midbrain.. Exp Cell Res.

[36] Bryja V, Gradl D, Schambony A, Arenas E, Schulte G (2007). beta-arrestin is a necessary component of Wnt/beta-catenin signaling in vitro and in vivo.. Proc Natl Acad Sci USA.

[37] Rawal N, Castelo-Branco G, Sousa KM, Kele J, Kobayashi K (2006). Dynamic temporal and cell type-specific expression of Wnt signaling components in the developing midbrain.. Exp Cell Res.

[38] Yamaguchi TP, Bradley A, McMahon AP, Jones S (1999). A Wnt5a pathway underlies outgrowth of multiple structures in the vertebrate embryo.. Development.

[39] Nemeth MJ, Topol L, Anderson SM, Yang Y, Bodine DM (2007). Wnt5a inhibits canonical Wnt signaling in hematopoietic stem cells and enhances repopulation.. Proc Natl Acad Sci U S A.

[40] Prakash N, Brodski C, Naserke T, Puelles E, Gogoi R (2006). A Wnt1-regulated genetic network controls the identity and fate of midbrain-dopaminergic progenitors in vivo.. Development.

[41] Smidt MP, Smits SM, Bouwmeester H, Hamers FP, van der Linden AJ (2004). Early developmental failure of substantia nigra dopamine neurons in mice lacking the homeodomain gene Pitx3.. Development.

[42] van den Munckhof P, Luk KC, Ste-Marie L, Montgomery J, Blanchet PJ (2003). Pitx3 is required for motor activity and for survival of a subset of midbrain dopaminergic neurons.. Development.

[43] Nunes I, Tovmasian LT, Silva RM, Burke RE, Goff SP (2003). Pitx3 is required for development of substantia nigra dopaminergic neurons.. Proc Natl Acad Sci U S A.

[44] Prakash N, Wurst W (2006). Genetic networks controlling the development of midbrain dopaminergic neurons.. J Physiol.

[45] Kele J, Simplicio N, Ferri AL, Mira H, Guillemot F (2006). Neurogenin 2 is required for the development of ventral midbrain dopaminergic neurons.. Development.

[46] Andersson E, Tryggvason U, Deng Q, Friling S, Alekseenko Z (2006). Identification of intrinsic determinants of midbrain dopamine neurons.. Cell.

[47] Miyoshi G, Bessho Y, Yamada S, Kageyama R (2004). Identification of a novel basic helix-loop-helix gene, Heslike, and its role in GABAergic neurogenesis.. J Neurosci.

[48] Nakatani T, Minaki Y, Kumai M, Ono Y (2007). Helt determines GABAergic over glutamatergic neuronal fate by repressing Ngn genes in the developing mesencephalon.. Development.

[49] Parish CL, Castelo-Branco G, Rawal N, Tonnesen J, Sorensen AT (2008). Wnt5a-treated midbrain neural stem cells improve dopamine cell replacement therapy in parkinsonian mice.. J Clin Invest.

[50] Murdoch JN, Henderson DJ, Doudney K, Gaston-Massuet C, Phillips HM (2003). Disruption of scribble (Scrb1) causes severe neural tube defects in the circletail mouse.. Hum Mol Genet.

[51] Kibar Z, Vogan KJ, Groulx N, Justice MJ, Underhill DA (2001). Ltap, a mammalian homolog of Drosophila Strabismus/Van Gogh, is altered in the mouse neural tube mutant Loop-tail.. Nat Genet.

[52] Greene ND, Gerrelli D, Van Straaten HW, Copp AJ (1998). Abnormalities of floor plate, notochord and somite differentiation in the loop-tail (Lp) mouse: a model of severe neural tube defects.. Mech Dev.

[53] Lien WH, Klezovitch O, Fernandez TE, Delrow J, Vasioukhin V (2006). alphaE-catenin controls cerebral cortical size by regulating the hedgehog signaling pathway.. Science.

[54] Castelo-Branco G, Sousa KM, Bryja V, Pinto L, Wagner J (2006). Ventral midbrain glia express region-specific transcription factors and regulate dopaminergic neurogenesis through Wnt-5a secretion.. Mol Cell Neurosci.

[55] Copp AJ, Greene ND, Murdoch JN (2003). The genetic basis of mammalian neurulation.. Nat Rev Genet.

[56] Veeman MT, Axelrod JD, Moon RT (2003). A second canon. Functions and mechanisms of beta-catenin-independent Wnt signaling.. Dev Cell.

[57] Bush KT, Lynch FJ, DeNittis AS, Steinberg AB, Lee HY (1990). Neural tube formation in the mouse: a morphometric and computerized three-dimensional reconstruction study of the relationship between apical constriction of neuroepithelial cells and the shape of the neuroepithelium.. Anat Embryol (Berl).

[58] Copp AJ, Greene ND, Murdoch JN (2003). Dishevelled: linking convergent extension with neural tube closure.. Trends Neurosci.

[59] Shariatmadari M, Peyronnet J, Papachristou P, Horn Z, Sousa KM (2005). Increased Wnt levels in the neural tube impair the function of adherens junctions during neurulation.. Mol Cell Neurosci.

[60] Suzuki A, Yamanaka T, Hirose T, Manabe N, Mizuno K (2001). Atypical protein kinase C is involved in the evolutionarily conserved par protein complex and plays a critical role in establishing epithelia-specific junctional structures.. J Cell Biol.

[61] Ciruna B, Jenny A, Lee D, Mlodzik M, Schier AF (2006). Planar cell polarity signalling couples cell division and morphogenesis during neurulation.. Nature.

[62] Gong Y, Mo C, Fraser SE (2004). Planar cell polarity signalling controls cell division orientation during zebrafish gastrulation.. Nature.

[63] Wu M, Herman MA (2006). A novel noncanonical Wnt pathway is involved in the regulation of the asymmetric B cell division in C. elegans.. Dev Biol.

[64] Conlon RA, Herrmann BG (1993). Detection of messenger RNA by in situ hybridization to postimplantation embryo whole mounts.. Methods Enzymol.

[65] Brodski C, Weisenhorn DM, Signore M, Sillaber I, Oesterheld M (2003). Location and size of dopaminergic and serotonergic cell populations are controlled by the position of the midbrain-hindbrain organizer.. J Neurosci.

[66] Puelles E, Annino A, Tuorto F, Usiello A, Acampora D (2004). Otx2 regulates the extent, identity and fate of neuronal progenitor domains in the ventral midbrain.. Development.

[67] Puelles E, Acampora D, Lacroix E, Signore M, Annino A (2003). Otx dose-dependent integrated control of antero-posterior and dorso-ventral patterning of midbrain.. Nat Neurosci.

[68] Cau E, Gradwohl G, Fode C, Guillemot F (1997). Mash1 activates a cascade of bHLH regulators in olfactory neuron progenitors.. Development.

[69] Goodrich LV, Johnson RL, Milenkovic L, McMahon JA, Scott MP (1996). Conservation of the hedgehog/patched signaling pathway from flies to mice: induction of a mouse patched gene by Hedgehog.. Genes Dev.

[70] Hui CC, Slusarski D, Platt KA, Holmgren R, Joyner AL (1994). Expression of three mouse homologs of the Drosophila segment polarity gene cubitus interruptus, Gli, Gli-2, and Gli-3, in ectoderm- and mesoderm-derived tissues suggests multiple roles during postimplantation development.. Dev Biol.

[71] Guimera J, Weisenhorn DV, Wurst W (2006). Megane/Heslike is required for normal GABAergic differentiation in the mouse superior colliculus.. Development.

