# Supplementary figures and images for: Wnt5a Regulates Ventral Midbrain Morphogenesis and the Development of A9–A10 Dopaminergic Cells In Vivo
Source: PLoS One. 2008 Oct 27;3(10):e3517. doi: 10.1371/journal.pone.0003517 (PMC2568809; doi:10.1371/journal.pone.0003517)

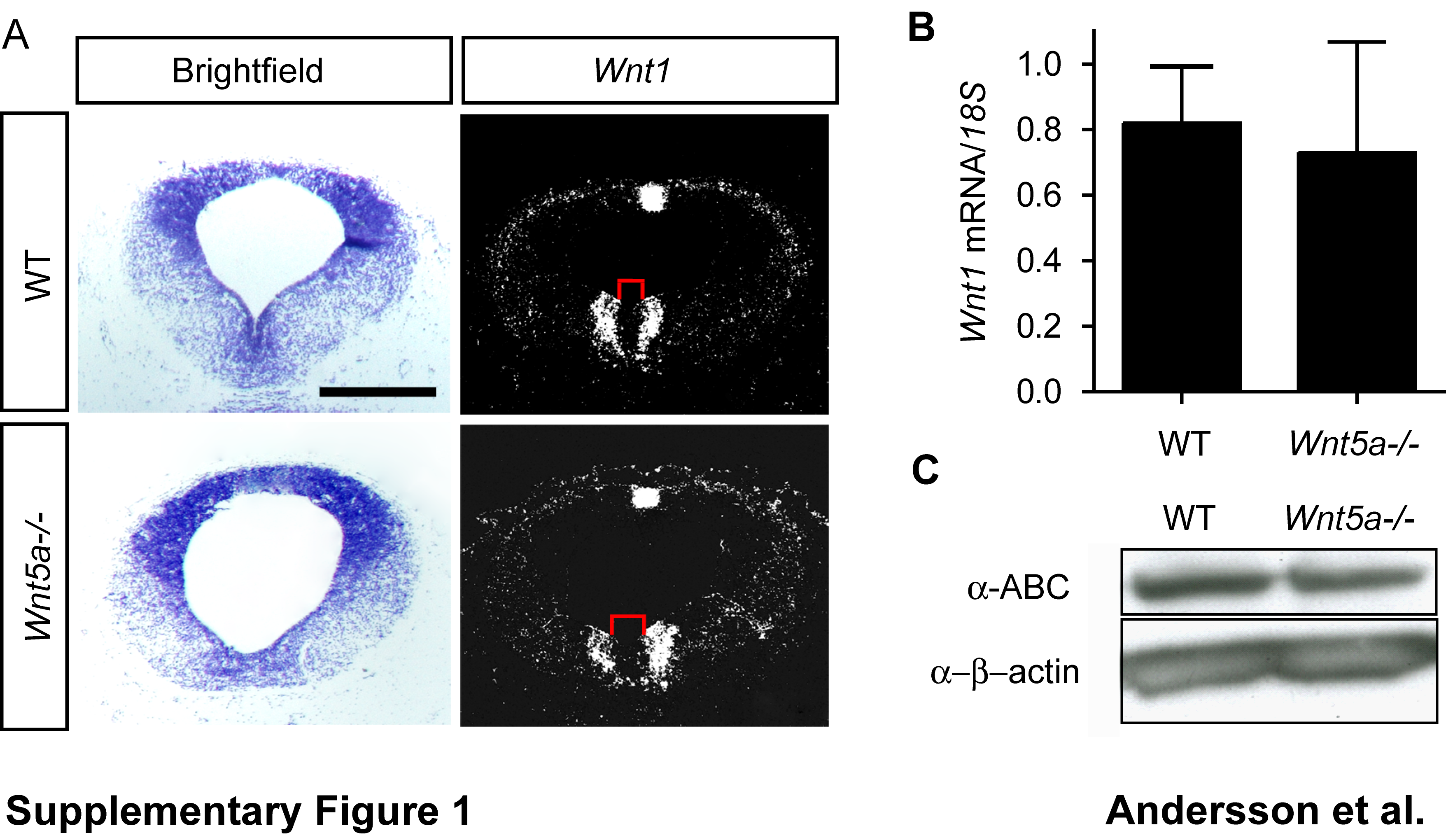

Supplement: Figure S1 — Canonical Wnt signaling is unaffected by loss of Wnt5a. (A) In situ hybridization at E12.5 for Wnt1 shows wider spacing between the two ventral stripes of Wnt1 expression in the Wnt5a−/− mice (red brackets), but no enlargement of the Wnt1 domain (Scale bar is 500 µm). (B) QPCR for Wnt1 shows no difference in levels of Wnt1 in the VM of Wnt5a−/− mice at E12.5. (C) Western blot for active (dephosphorylated) β-catenin shows little difference at E12.5 in the Wnt5a−/− VM. (4.57 MB TIF) [file pone.0003517.s001.tif]

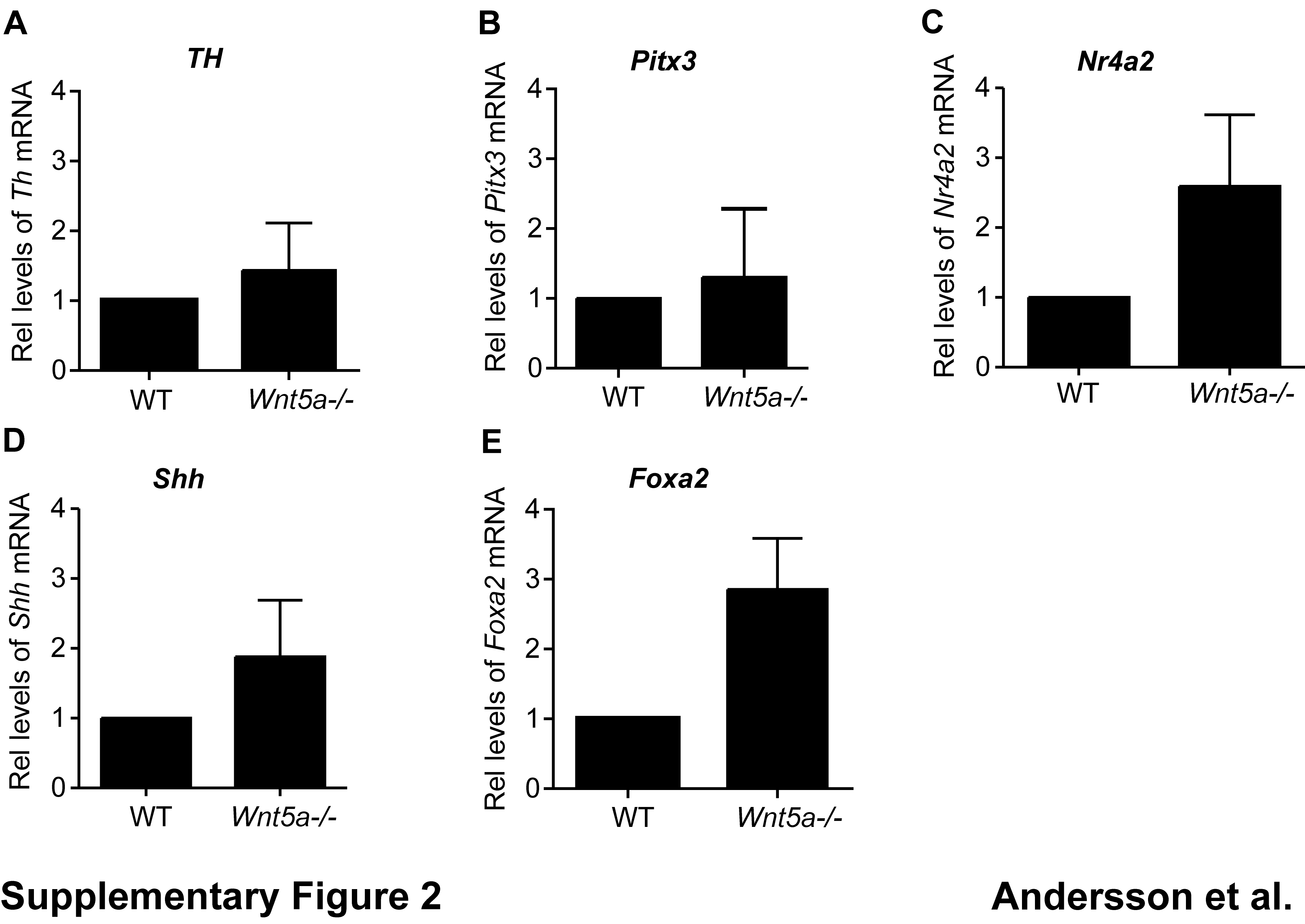

Supplement: Figure S2 — QPCR of E12.5 WT and Wnt5a−/− VM. No difference in Th (A) and Pitx3 (B) mRNA levels. Increase in the amount of Nr4a2 mRNA (C). Small or no increase in Shh mRNA (D) and a greater difference in Foxa2 levels (E). (0.71 MB TIF) [file pone.0003517.s002.tif]

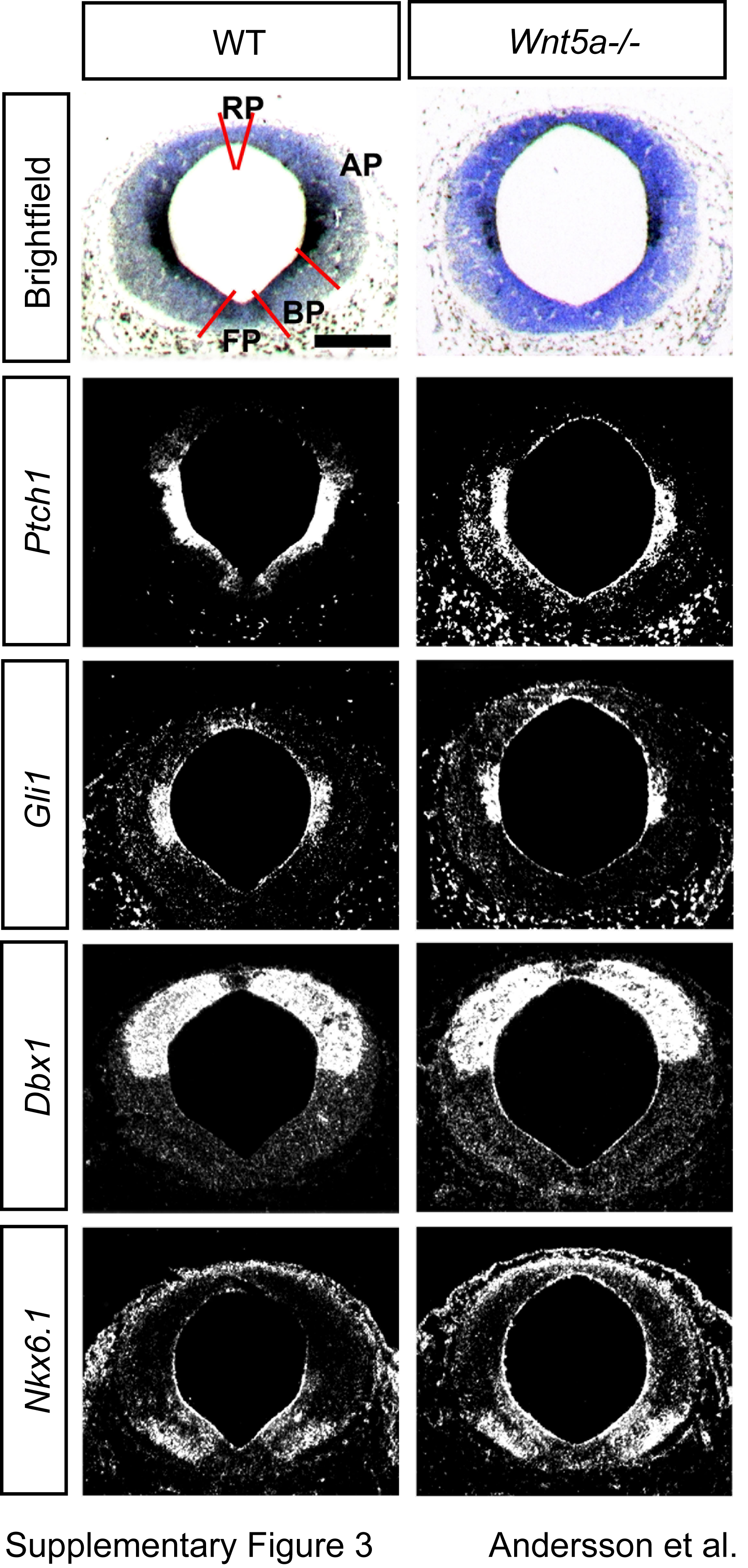

Supplement: Figure S3 — No change in dorsoventral patterning of the Wnt5a−/− midbrain. Expression of Shh-target genes Ptch1 and Gli1 was not altered in Wnt5a−/− mice. The expression of class I (Dbx1) and class II (Nkx6-1) genes was not changed in the midbrain of the Wnt5a−/− embryos compared to WT. (5.40 MB TIF) [file pone.0003517.s003.tif]

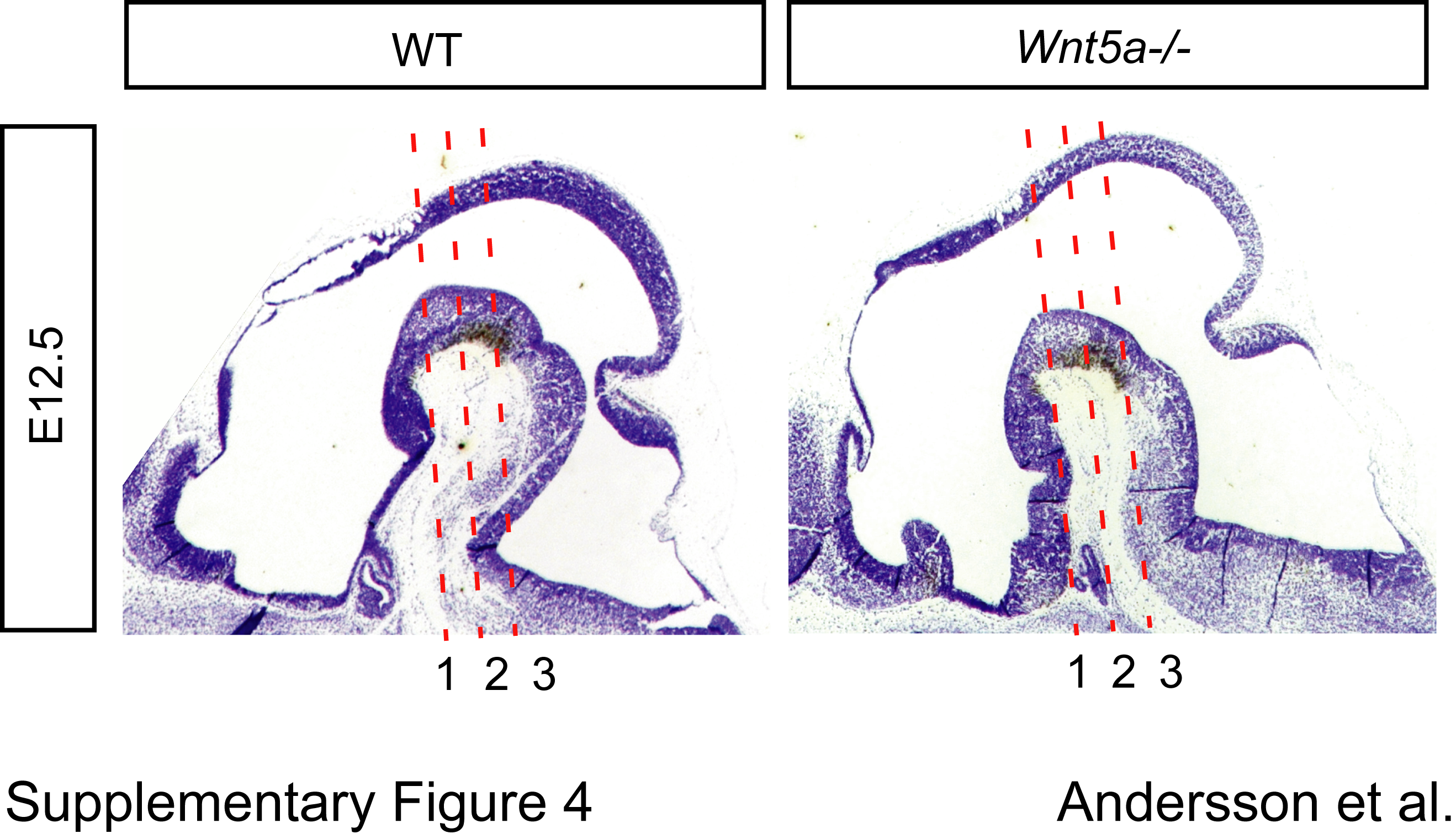

Supplement: Figure S4 — Anteroposterior levels used to analyze the lateral distribution of TH+ cells. Levels 1, 2 and 3 corresponding to rostral, intermediate and caudal levels are depicted on sagittal sections of WT and Wnt5a−/− mice, probed for Th. (3.81 MB TIF) [file pone.0003517.s004.tif]

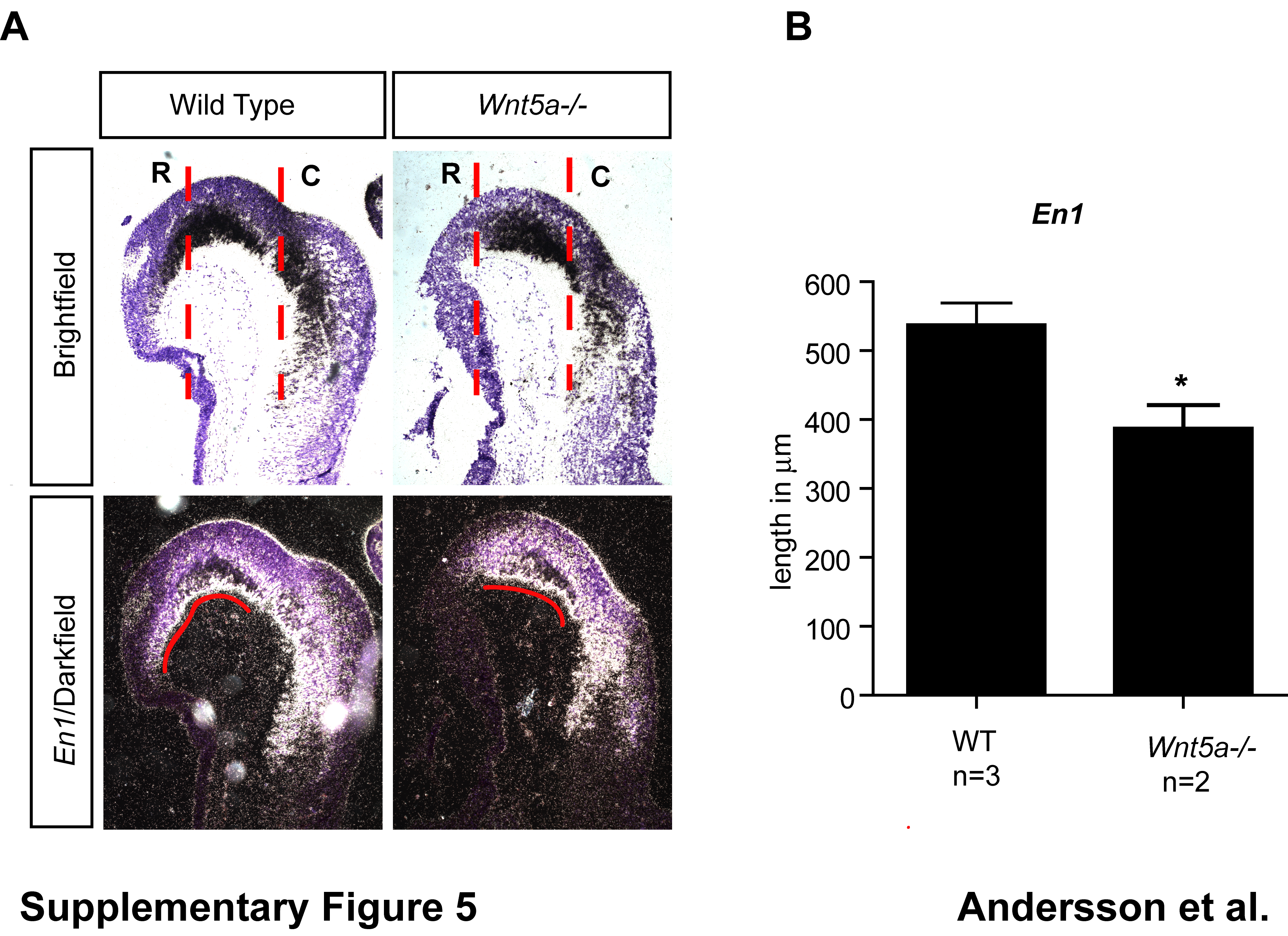

Supplement: Figure S5 — The En1-expressing domain is shortened in Wnt5a−/− VM (A) Bright field and dark field images of the midbrain cephalic flexure hybridized for Engrailed1 (En1) show a shorter anteroposterior extension of En1 in the Wnt5a−/− midbrain. Red line shows the length measured from isthmus to anterior-most En1 expression. (B) Quantification of En1 expression length shows a shorter domain in Wnt5a−/− embryos (paired t-test p = 0.0203, WT N = 3, Wnt5a−/− N = 2). (7.93 MB TIF) [file pone.0003517.s005.tif]
